# The genome sequence of the European hornet, *Vespa crabro *Linnaeus, 1758

**DOI:** 10.12688/wellcomeopenres.17546.1

**Published:** 2022-01-27

**Authors:** Liam M. Crowley, University of Oxford and Wytham Woods Genome Acquisition Lab, Darwin Tree of Life Barcoding collective, Wellcome Sanger Institute Tree of Life programme, Wellcome Sanger Institute Scientific Operations: DNA Pipelines collective, Tree of Life Core Informatics collective, Darwin Tree of Life Consortium

**Affiliations:** 1 Department of Zoology, University of Oxford, Oxford, UK

**Keywords:** Vespa crabro, European hornet, genome sequence, chromosomal, Hymenoptera

## Abstract

We present a genome assembly from an individual female *Vespa crabro *(the European hornet; Arthropoda; Insecta; Hymenoptera; Vespidae). The genome sequence is 230 megabases in span. The majority of the assembly (94.93%) is scaffolded into 25 chromosomal pseudomolecules.

## Species taxonomy

Eukaryota; Metazoa; Ecdysozoa; Arthropoda; Hexapoda; Insecta; Pterygota; Neoptera; Endopterygota; Hymenoptera; Apocrita; Aculeata; Vespoidea; Vespidae; Vespinae; Vespa; *Vespa crabro* Linnaeus, 1758 (NCBI:txid7445).

## Background

The European hornet, *Vespa crabro*, is the largest social wasp in Europe, with workers measuring up to 23 mm and queens up to 30 mm ( Archer & Turner, 2014). It is common and widespread throughout much of the palearctic, including throughout England and Wales, and has been introduced into North America ( Archer, 1992). *Vespa crabro* occurs in a wide range of habitats but is particularly associated with mature deciduous woodlands and urban environments. The head and majority of the metasoma are predominantly yellow and the mesosoma, anterior segments of the metasoma and legs are reddish-brown with varying extents of black markings.

This species is eusocial, living in colonies with a queen, workers and males. Whilst workers are capable of producing male offspring, this is rare due to the action of worker policing ( Foster *et al.*, 2002). Colonies are founded by overwintered queens from early April, with the first workers appearing in late June to early July ( Bunn, 1988b). Nests are constructed out of a paper-like substance produced from the pulp of decaying wood mixed with saliva, often incorporating bark and twig fragments ( Spredbery, 1973). The nest consists of up to around 1400 hexagonal cells arranged into 3-8 combs, covered by a nest envelope ( Archer, 1993; Archer, 2008). Nests are usually located in aerial situations, particularly in hollow trees, but are also commonly located in human structures such as attics and outbuildings. Colonies may relocate the nest should it exceed the available space ( Pawlyszyn, 1992). Nests are occasionally located underground, but such nests are more frequently relocated ( Archer & Turner, 2014). The nests are well characterised as containing many species of inquilines, predators and parasitoids. Colonies build up throughout the summer, before the reproductive males and gynes are produced in September. Most queens are singularly mated but double and triple mating also occurs, although paternity of the offspring produced by multiply-mated queens is heavily biased to a single male ( Foster *et al.*, 1999). Workers may persist until October or occasionally early November ( Archer, 1993; Bunn, 1988b).

Female hornets are generalist predators, catching, killing, and preparing prey of various arthropods to take back to the nest to feed to the developing brood. Prey includes other species of social wasp, honeybees, flies, butterflies, moths and spiders ( Pawlyszyn, 1994). In particular, returning honeybee foragers are frequently taken, although this species does not inflict a considerable impact on honeybee colonies due to honeybee defensive behaviours ( Baracchi *et al.*, 2010). *Vespa crabro* also often frequents sap runs, where it feeds on the exudations, and is also known to rig bark twigs to stimulate sap flow ( Bunn, 1988a; Edwards, 1980). Ivy ( *Hedera helix*) flowers are visited when in bloom to feed on nectar ( Pawlyszyn, 1994), particularly by males. The trophic preferences of this species overlap with the invasive *Vespa velutina*, meaning that where these species co-occur, *V. crabro* may be outperformed in instances of interspecific competition ( Cini *et al.*, 2018).

The species is not particularly aggressive, although females may sting if provoked or in defence of the nest. Major venom components, such as prepromastoparan, vespid chemotactic peptide precursor and vespakinin, are more highly enriched that in some other species of *Vespa*, meaning that the venom likely has a greater toxicity ( Yoon *et al.*, 2015).

## Genome sequence report

**Figure 1.**
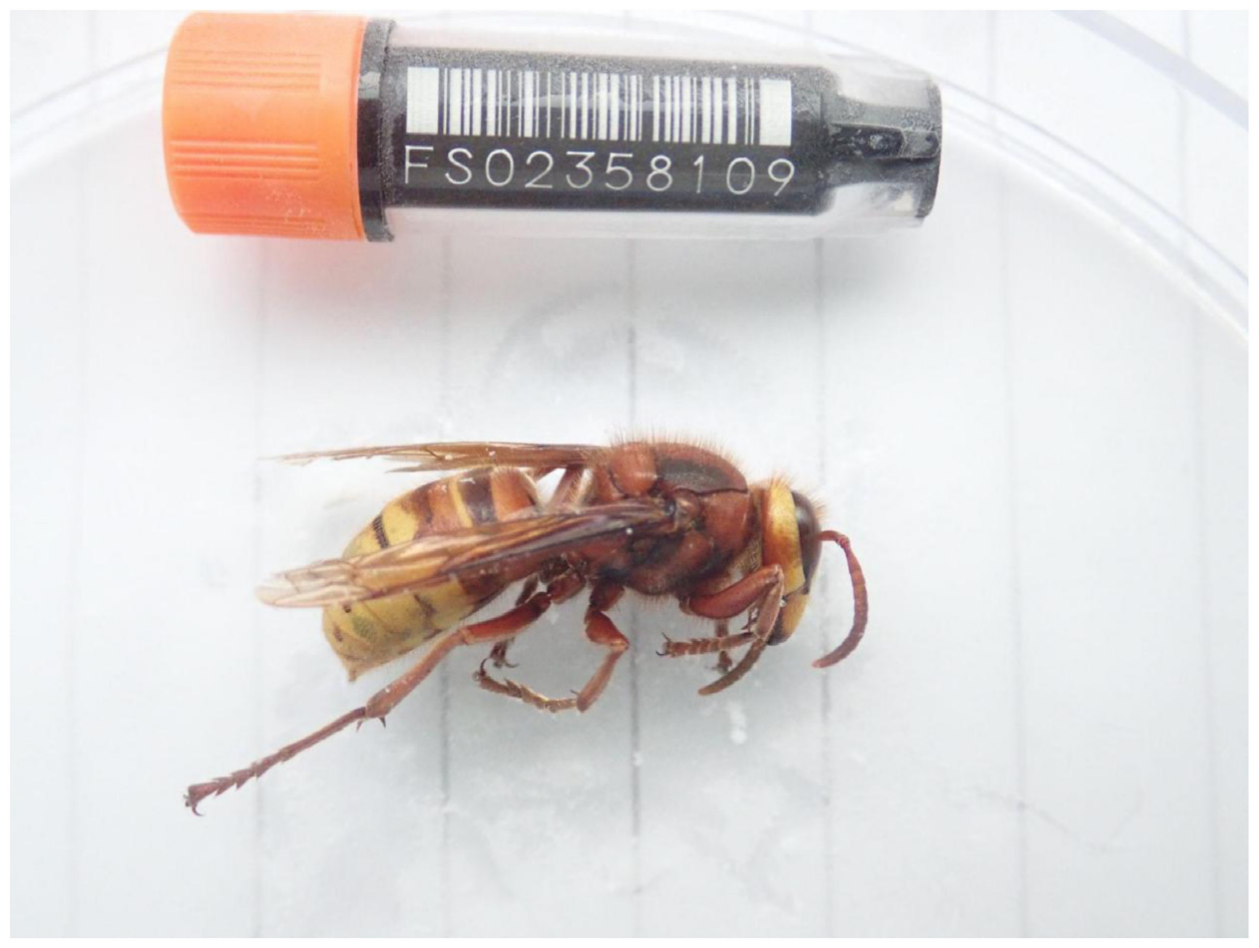
Image of the iyVesCrab1 specimen taken during preservation and processing.

The genome was sequenced from a single female *V. crabro* ( Figure 1) collected from Wytham Woods, Oxfordshire, UK (latitude 51.77, longitude -1.338). A total of 70-fold coverage in Pacific Biosciences single-molecule long reads and 104-fold coverage in 10X Genomics read clouds were generated. Primary assembly contigs were scaffolded with chromosome conformation Hi-C data. Manual assembly curation corrected 96 missing/misjoins, reducing the scaffold number by 41.67%, and increasing the scaffold N50 by 113.35% and assembly length by 0.01%.

**Figure 2.**
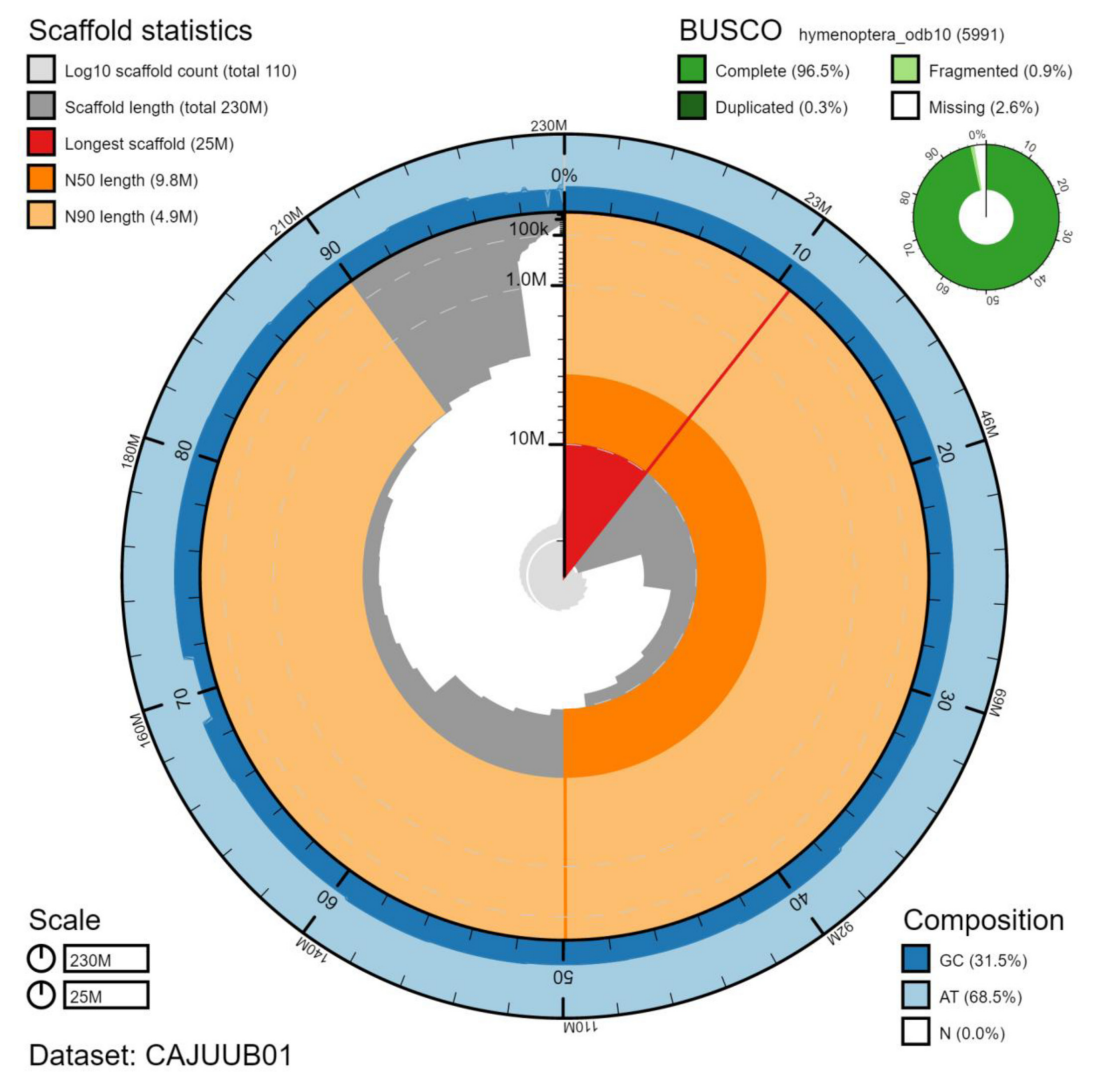
Genome assembly of *Vespa crabro*, iyVesCrab1: metrics. The BlobToolKit Snailplot shows N50 metrics and BUSCO gene completeness. The main plot is divided into 1,000 size-ordered bins around the circumference with each bin representing 0.1% of the 229,601,916 bp assembly. The distribution of scaffold lengths is shown in dark grey with the plot radius scaled to the longest scaffold present in the assembly (24,517,513 bp, shown in red). Orange and pale-orange arcs show the N50 and N90 chromosome lengths (9,767,562 and 4,871,713 bp), respectively. The pale grey spiral shows the cumulative chromosome count on a log scale with white scale lines showing successive orders of magnitude. The blue and pale-blue area around the outside of the plot shows the distribution of GC, AT and N percentages in the same bins as the inner plot. A summary of complete, fragmented, duplicated and missing BUSCO genes in the hymenoptera_odb10 set is shown in the top right. An interactive version of this figure is available at https://blobtoolkit.genomehubs.org/view/iyVesCrab1.1/dataset/CAJUUB01/snail.

The final assembly has a total length of 230 Mb in 106 sequence scaffolds with a scaffold N50 of 10 Mb ( Table 1). Of the assembly sequence, 94.9% was assigned to 25 chromosomal-level scaffolds (numbered by sequence length) ( Figure 2– Figure 5; Table 2). The assembly has a BUSCO v5.1.2 ( Simão *et al.*, 2015) completeness of 96.5% (single 96.3%, duplicated 0.3%) using the hymenoptera_odb10 reference set. While not fully phased, the assembly deposited is of one haplotype. Contigs corresponding to the second haplotype have also been deposited.

**Table 1.** Genome data for *Vespa crabro*, iyVesCrab1.1.

*Project accession data*	
Assembly identifier	iyVesCrab1.1
Species	*Vespa crabro*
Specimen	iyVesCrab1
NCBI taxonomy ID	NCBI:txid7445
BioProject	PRJEB45175
BioSample ID	SAMEA7520500
Isolate information	Female, head/thorax/abdomen
*Raw data accessions*	
PacificBiosciences SEQUEL II	ERR6406214, ERR6608657, ERR6608658
10X Genomics Illumina	ERR6054851-ERR6054858
Hi-C Illumina	ERR6054859
*Genome assembly*	
Assembly accession	GCA_910589235.1
*Accession of alternate haplotype*	GCA_910589515.1
Span (Mb)	230
Number of contigs	213
Contig N50 length (Mb)	3.0
Number of scaffolds	105
Scaffold N50 length (Mb)	9.8
Longest scaffold (Mb)	24.5
BUSCO * genome score	C:96.5%[S:96.3%,D:0.3%],F:0.9%,M:2.6%,n:5991

*BUSCO scores based on the hymenoptera_odb10 BUSCO set using v5.1.2. C= complete [S= single copy, D=duplicated], F=fragmented, M=missing, n=number of orthologues in comparison. A full set of BUSCO scores is available at https://blobtoolkit.genomehubs.org/view/iyVesCrab1.1/dataset/CAJUUB01/busco.

**Figure 3.**
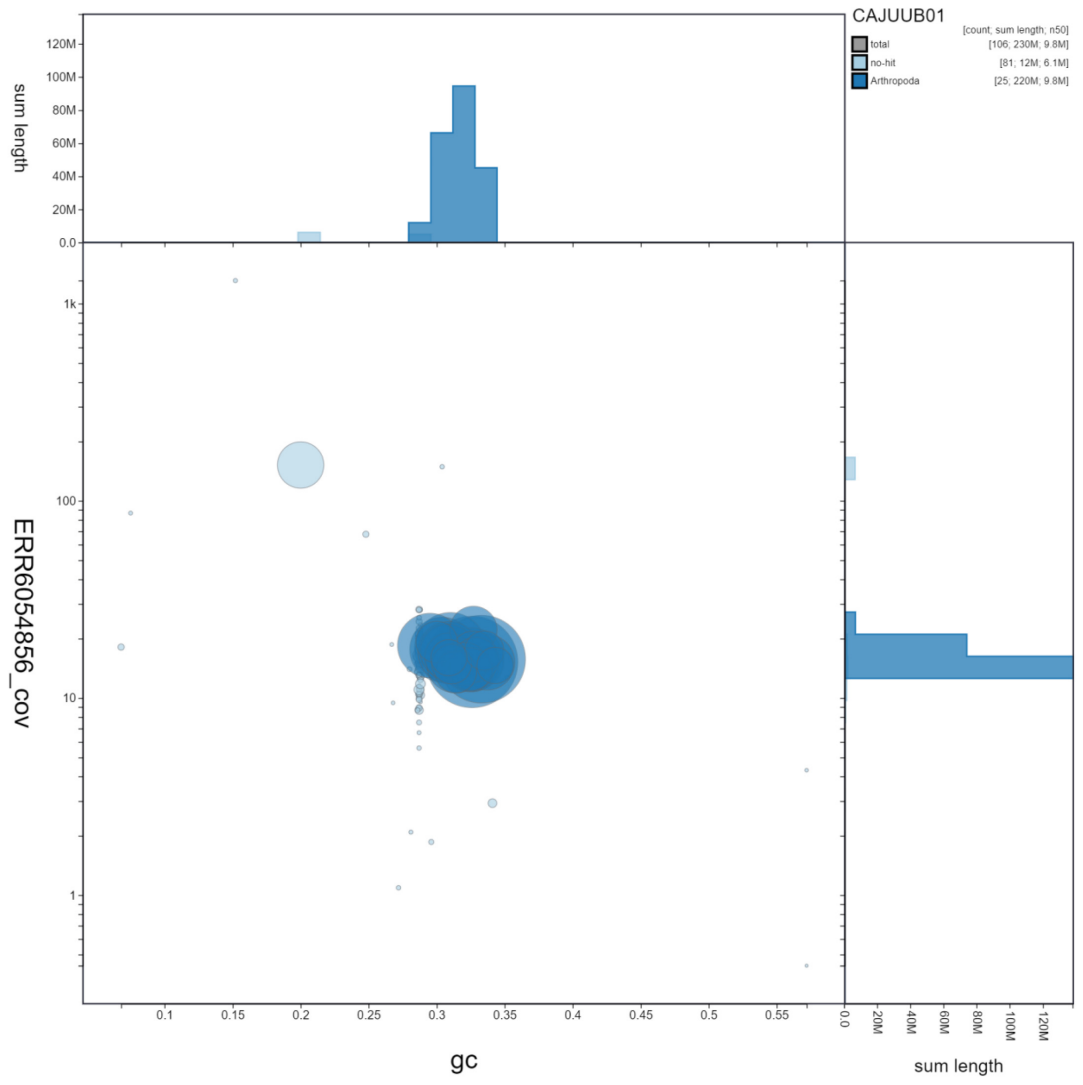
Genome assembly of *Vespa crabro*, iyVesCrab1: GC coverage. BlobToolKit GC-coverage plot. Scaffolds are coloured by phylum. Circles are sized in proportion to scaffold length. Histograms show the distribution of scaffold length sum along each axis. An interactive version of this figure is available at https://blobtoolkit.genomehubs.org/view/iyVesCrab1.1/dataset/CAJUUB01/blob.

**Figure 4.**
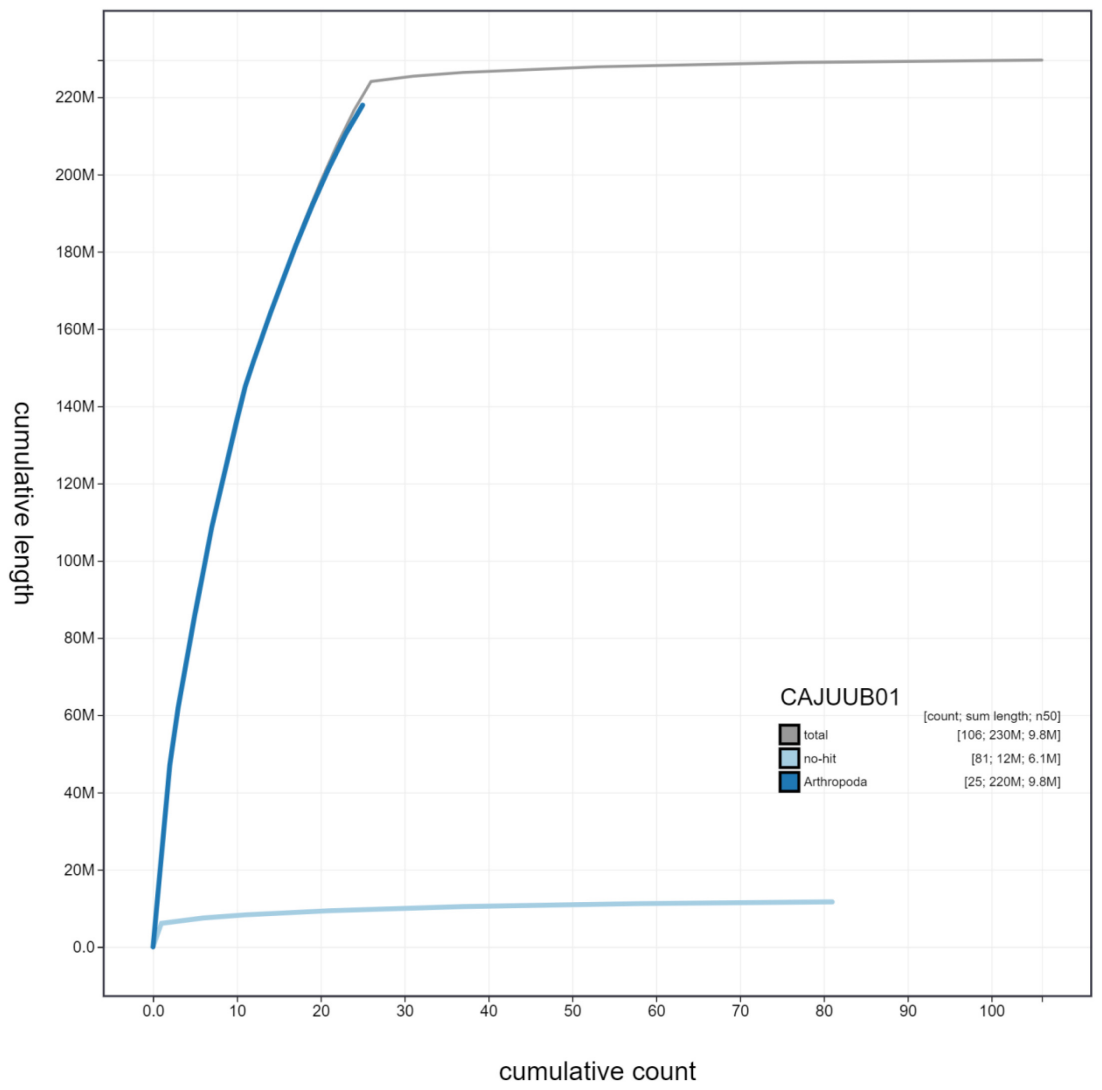
Genome assembly of *Vespa crabro*, iyVesCrab1: cumulative sequence. BlobToolKit cumulative sequence plot. The grey line shows cumulative length for all scaffolds. Coloured lines show cumulative lengths of scaffolds assigned to each phylum using the buscogenes taxrule. An interactive version of this figure is available at https://blobtoolkit.genomehubs.org/view/iyVesCrab1.1/dataset/CAJUUB01/cumulative.

**Figure 5.**
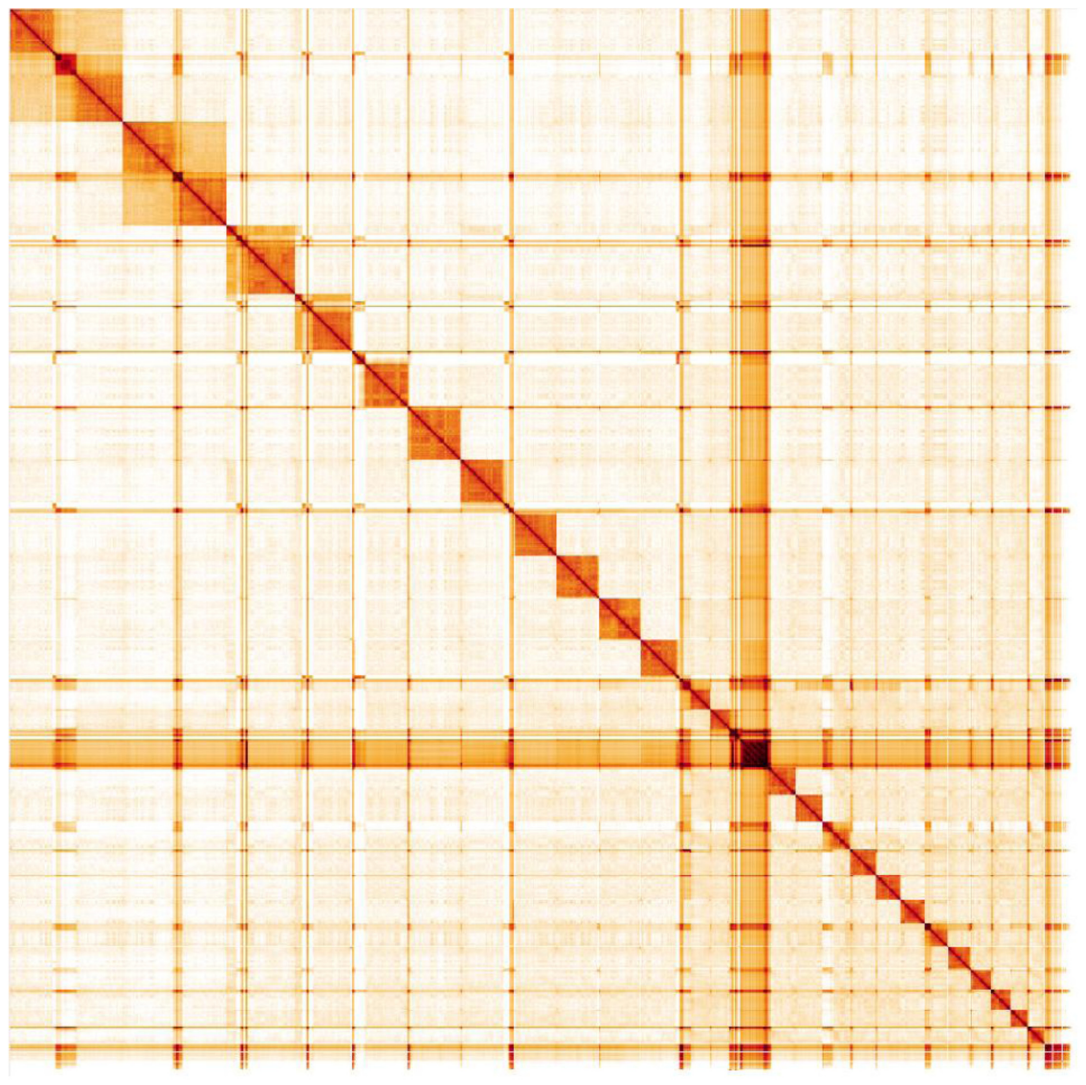
Genome assembly of *Vespa crabro*, iyVesCrab1: Hi-C contact map. Hi-C contact map of the iyVesCrab1.1 assembly, visualised in HiGlass. Chromosomes are shown in size order from left to right and top to bottom.

The assembly was constructed into 25 chromosomes and seems in agreement with the expected karyotype from Hoshiba *et al.* (1989). Large centromeric regions were observed in the Hi-C map, notably on SUPER_1, SUPER_12 and SUPER_16 (~2-3 Mbp in size), and could explain the skew in genome content to AT. Scaffold_5 (~6 Mbp in size) appears to be a collapsed centromeric repeat and could not be confidently placed.

**Table 2.** Chromosomal pseudomolecules in the genome assembly of *Vespa crabro*, iyVesCrab1.1.

INSDC accession	Chromosome	Size (Mb)	GC%
OU342400.1	1	24.52	32.6
OU342401.1	2	22.54	33.3
OU342402.1	3	14.88	31.0
OU342403.1	4	12.12	30.4
OU342404.1	5	12.04	29.5
OU342405.1	6	11.60	32.6
OU342406.1	7	10.99	30.9
OU342407.1	8	9.77	31.4
OU342408.1	9	9.21	32.5
OU342409.1	10	8.84	33.7
OU342410.1	11	8.62	30.8
OU342411.1	12	6.64	30.9
OU342412.1	13	6.37	32.7
OU342413.1	14	5.95	32.1
OU342414.1	15	5.84	32.6
OU342415.1	16	5.79	32.7
OU342416.1	17	5.72	32.9
OU342417.1	18	5.29	32.1
OU342418.1	19	5.27	32.5
OU342419.1	20	4.92	30.0
OU342420.1	21	4.87	31.4
OU342421.1	22	4.45	31.1
OU342422.1	23	4.32	33.5
OU342423.1	24	3.72	34.3
OU342424.1	25	3.65	30.9
OU342425.1	MT	0.02	15.2
-	Unplaced	11.64	24.1

## Methods

### Sample acquisition and DNA extraction

A single female *V. crabro* was collected from Wytham Woods, Oxfordshire, UK (latitude 51.774, longitude -1.332) by Liam Crowley, University of Oxford, using a net. The sample was identified by the same individual and snap-frozen on dry ice.

DNA was extracted at the Tree of Life laboratory, Wellcome Sanger Institute. The iyVesCrab1 sample was weighed and dissected on dry ice with tissue set aside for Hi-C sequencing. Thorax tissue was disrupted using a Nippi Powermasher fitted with a BioMasher pestle. Fragment size analysis of 0.01-0.5 ng of DNA was then performed using an Agilent FemtoPulse. High molecular weight (HMW) DNA was extracted using the Qiagen MagAttract HMW DNA extraction kit. Low molecular weight DNA was removed from a 200-ng aliquot of extracted DNA using 0.8X AMpure XP purification kit prior to 10X Chromium sequencing; a minimum of 50 ng DNA was submitted for 10X sequencing. HMW DNA was sheared into an average fragment size between 12-20 kb in a Megaruptor 3 system with speed setting 30. Sheared DNA was purified by solid-phase reversible immobilisation using AMPure PB beads with a 1.8X ratio of beads to sample to remove the shorter fragments and concentrate the DNA sample. The concentration of the sheared and purified DNA was assessed using a Nanodrop spectrophotometer and Qubit Fluorometer and Qubit dsDNA High Sensitivity Assay kit. Fragment size distribution was evaluated by running the sample on the FemtoPulse system.

### Sequencing

Pacific Biosciences HiFi circular consensus and 10X Genomics read cloud sequencing libraries were constructed according to the manufacturers’ instructions. Sequencing was performed by the Scientific Operations core at the Wellcome Sanger Institute on Pacific Biosciences SEQUEL II and Illumina HiSeq X instruments. Hi-C data were generated from remaining thorax tissue using the Arima v2.0 kit and sequenced on an Illumina NovaSeq 6000 instrument.

### Genome assembly

Assembly was carried out with Hifiasm ( Cheng *et al.*, 2021). Haplotypic duplication was identified and removed with purge_dups ( Guan *et al.*, 2020). Scaffolding with Hi-C data ( Rao *et al.*, 2014) was carried out with SALSA2 ( Ghurye *et al.*, 2019). The Hi-C scaffolded assembly was polished with the 10X Genomics Illumina data by aligning to the assembly with longranger align, calling variants with freebayes ( Garrison & Marth, 2012). One round of the Illumina polishing was applied. The mitochondrial genome was assembled with MitoHiFi ( Uliano-Silva *et al.*, 2021), which performed annotation using MitoFinder ( Allio *et al.*, 2020). The assembly was checked for contamination and corrected using the gEVAL system ( Chow *et al.*, 2016) as described previously ( Howe *et al.*, 2021). Manual curation ( Howe *et al.*, 2021) was performed using gEVAL, HiGlass ( Kerpedjiev *et al.*, 2018) and Pretext. The genome was analysed within the BlobToolKit environment ( Challis *et al.*, 2020). Table 3 contains a list of all software tool versions used, where appropriate.

**Table 3.** Software tools used.

Software tool	Version	Source
Hifiasm	0.12	Cheng *et al.*, 2021
purge_dups	1.2.3	Guan *et al.*, 2020
SALSA2	2.2	Ghurye *et al.*, 2019
longranger align	2.2.2	https://support.10xgenomics.com/ genome-exome/software/pipelines/latest/ advanced/other-pipelines
freebayes	v1.3.1-17- gaa2ace8	Garrison & Marth, 2012
MitoHiFi	1.0	Uliano-Silva *et al*. (2021)
gEVAL	N/A	Chow *et al.*, 2016
HiGlass	1.11.6	Kerpedjiev *et al.*, 2018
PretextView	0.0.4	https://github.com/wtsi-hpag/PretextView
BlobToolKit	2.6.4	Challis *et al.*, 2020

### Ethics/compliance issues

The materials that have contributed to this genome note have been supplied by a Darwin Tree of Life Partner. The submission of materials by a Darwin Tree of Life Partner is subject to the Darwin Tree of Life Project Sampling Code of Practice. By agreeing with and signing up to the Sampling Code of Practice, the Darwin Tree of Life Partner agrees they will meet the legal and ethical requirements and standards set out within this document in respect of all samples acquired for, and supplied to, the Darwin Tree of Life Project. Each transfer of samples is further undertaken according to a Research Collaboration Agreement or Material Transfer Agreement entered into by the Darwin Tree of Life Partner, Genome Research Limited (operating as the Wellcome Sanger Institute), and in some circumstances other Darwin Tree of Life collaborators.

## Data availability

European Nucleotide Archive: Vespa crabro (European hornet). Accession number PRJEB45175: https://identifiers.org/ena.embl:PRJEB45175

The genome sequence is released openly for reuse. The *V. crabro* genome sequencing initiative is part of the Darwin Tree of Life (DToL) project. All raw sequence data and the assembly have been deposited in INSDC databases. The genome will be annotated and presented through the Ensembl pipeline at the European Bioinformatics Institute. Raw data and assembly accession identifiers are reported in Table 1.

## Author information

Members of the University of Oxford and Wytham Woods Genome Acquisition Lab are listed here: https://doi.org/10.5281/zenodo.5746938.

Members of the Darwin Tree of Life Barcoding collective are listed here: https://doi.org/10.5281/zenodo.5744972.

Members of the Wellcome Sanger Institute Tree of Life programme are listed here: https://doi.org/10.5281/zenodo.5744840.

Members of Wellcome Sanger Institute Scientific Operations: DNA Pipelines collective are listed here: https://doi.org/10.5281/zenodo.5746904.

Members of the Tree of Life Core Informatics collective are listed here: https://doi.org/10.5281/zenodo.5743293.

Members of the Darwin Tree of Life Consortium are listed here: https://doi.org/10.5281/zenodo.5638618.
