# Supplementary material for: The genome sequence of the European hornet, Vespa crabro Linnaeus, 1758
Source: Wellcome Open Res. 2022 Jan 27;7:27. [Version 1] doi: 10.12688/wellcomeopenres.17546.1 (PMC10514574; doi:10.12688/wellcomeopenres.17546.1)
Supplement: Peer review history

# Reviewer report: Reviewer response for version 1

**DOI:** 10.21956/wellcomeopenres.19402.r67215

**Published:** 2023-09-21

**Authors:** Barbara Feldmeyer

**Affiliations:** 1 Senckenberg Biodiversity and Climate Research Centre, Frankfurt am Main, Germany

This manuscript reports on the genome assembly of the European hornet, *Vespa crabro*. It is well-written and details most of the necessary information.

 The information on the sequencing library kit information should be added to the methods section. It might also be worthwhile spending some additional time on manually curating scaffolds.

Are sufficient details of methods and materials provided to allow replication by others?

Partly

Is the rationale for creating the dataset(s) clearly described?

Yes

Are the datasets clearly presented in a useable and accessible format?

Yes

Are the protocols appropriate and is the work technically sound?

Yes

Reviewer Expertise:

evolutionary biology, genomics

I confirm that I have read this submission and believe that I have an appropriate level of expertise to confirm that it is of an acceptable scientific standard.

# Reviewer report: Reviewer response for version 1

**DOI:** 10.21956/wellcomeopenres.19402.r48317

**Published:** 2022-02-18

**Authors:** Artem Nedoluzhko

**Affiliations:** 1 Russian Academy of Sciences, Saint Petersburg, Russian Federation

I thank the Wellcome Open Research authorities for handing me an opportunity to review a genome report: “The genome sequence of the European hornet, Vespa crabro Linnaeus, 1758”.

 The report of high-level assembly of European hornet genome is clearly presented and I have only minor comments to the authors:

1. You should use genus Vespa name only one time per manuscript text then use abbreviation *V. velutina *and *V. crabro.*
2. More detailed information about the invasion of *V. velutina *to Europe should be added (e.g. PMID: 33177635). ^1^
3. Explain why did you use a female hornet (a few words about sex determination in Hymenoptera)?
4. The place of specimen collection is presented twice in the report.
5. Please change citation for BUSCO v.5. ^2^

Are sufficient details of methods and materials provided to allow replication by others?

Yes

Is the rationale for creating the dataset(s) clearly described?

Yes

Are the datasets clearly presented in a useable and accessible format?

Yes

Are the protocols appropriate and is the work technically sound?

Yes

Reviewer Expertise:

evolutionary genomics, paleogenomics

I confirm that I have read this submission and believe that I have an appropriate level of expertise to confirm that it is of an acceptable scientific standard.

## References

[1] : Managing incursions of Vespa velutina nigrithorax in the UK: an emerging threat to apiculture. *Scientific Reports* .2020;10(1) : 10.1038/s41598-020-76690-2 10.1038/s41598-020-76690-2 33177635PMC7658989

[2] : BUSCO Update: Novel and Streamlined Workflows along with Broader and Deeper Phylogenetic Coverage for Scoring of Eukaryotic, Prokaryotic, and Viral Genomes. *Mol Biol Evol* .2021;38(10) : 10.1093/molbev/msab199 4647-4654 10.1093/molbev/msab199 34320186PMC8476166
